# Editorial: Racial and ethnic inequality in an age of populist nationalism

**DOI:** 10.3389/fsoc.2022.1026335

**Published:** 2022-09-15

**Authors:** Christel Kesler, Sin Yi Cheung

**Affiliations:** ^1^Department of Sociology, Colby College, Waterville, ME, United States; ^2^School of Social Sciences, Cardiff University, Cardiff, United Kingdom

**Keywords:** populist nationalism, race and ethnicity, inequalities, European societies, xenophobia

Populist nationalist politics–and concomitant racism, ethnocentrism, and xenophobia–have been on the rise in recent years in numerous countries around the globe. However, many questions remain about the context of racial and ethnic inequality in which populist nationalist sentiments take shape, and the direct consequences of populist nationalist sentiments, politics, and policies for racial and ethnic inequalities. This Research Topic brings together a collection of articles that shed new light on how contemporary populist nationalist politics are intertwined with on-the-ground experiences of racial and ethnic inequalities.

Two of the articles in the collection (Nandi and Luthra; Di Stasio and Heath) explore the experiences of ethnic minority groups in the aftermath of the Brexit vote in the United Kingdom, one of the most wellknown contemporary instances of populist nationalist politics. Nandi and Luthra focus on fear of and experiences of ethnic and racial harassment over the course of the Brexit vote, while Di Stasio and Heath examine discrimination in the labor market. With an explicit pre-/post-Brexit comparison, Nandi and Luthra find that fear of harassment clearly increased, especially among subgroups who had been less fearful in the past. Actual harassment, on the other hand, was mostly stable over time.

Di Stasio and Heath focus exclusively on the post-Brexit period and find through an audit study that both East and West European jobseekers generally face discrimination on the UK labor market, with the exception of West Europeans in the London metropolitan region. Even though Brexit specifically targeted European migrants, and especially East Europeans migrants, negative experiences in the wake of Brexit are not confined to European groups. Nandi and Luthra observe negative “spillover” effects among other ethnic minority groups. Indeed, non-white, non-European minority groups experience a greater increase in fear of ethnic and racial harassment, though intergroup differences are insignificant. Di Stasio and Heath do not include other minority groups in their analysis but note that the levels of discrimination they observe in their analysis of Europeans are lower than those documented among visible non-European minorities in other existing research.

Two of the other articles in the collection (Demireva and Zwysen; Kalter and Faroutan) reverse the causal arrow, to ask how various kinds of racial and ethnic inequalities affect the contours of populist nationalist sentiments. Demireva and Zwysen examine residential segregation and ask, using data from 16 Western European countries, how residence in local areas with a high migrant and minority concentration shapes both political sentiments and economic outcomes. Importantly, living in areas of minority concentration leads to higher rates of far-right voting and dissatisfaction with democracy among majority populations and also among the immigrant second generation. Living in an enclave also exacerbates economic inequalities, as it has a negative impact on employment outcomes for minority groups.

The analysis of Kalter and Faroutan examines anti-Muslim sentiment in Germany and demonstrates that as a result of their perceived lower status in German society, East Germans develop higher levels of anti-Muslim sentiment. In both of these papers, it is clear that contexts of greater perceived competition and threat, as we observe in ethnic enclaves across Europe and in the case of East Germans within the larger German society, have the potential to fuel populist nationalist politics.

The contribution by Kopyciok and Silver challenges a common assumption about xenophobia–that it is necessarily associated with the political right. Their analysis of left-wing xenophobia across 15 European countries shows that xenophobia is nearly as common on the political extreme left as it is on the extreme right, and less common among those with a more moderate political orientation. Xenophobic far-left individuals are socioeconomically disadvantaged compared to other groups, and they are more supportive of income redistribution and general egalitarianism, as we would expect from their left-wing self-placement. However, these desires for equality do not extend to immigrants. As on the xenophobic far right, welfare-chauvinist beliefs are far higher on the xenophobic far left than among the population at large. Indeed, the authors assert that if, in the future, politicians choose to cater to the concerns of the xenophobic far left, we may see high levels of redistribution but increasing levels of welfare chauvinism, undermining inclusive, universal welfare states where they existed, with obvious profound consequences for racial and ethnic inequalities in the future.

We would like to reflect briefly on this collection of papers and gaps in coverage in the collection that we hope will be filled by future research. First, we note that across nearly all of the papers in this Research Topic, local and regional contexts matter tremendously in shaping the way that populist nationalism manifests, whether that means thinking about where minority groups are most likely to experience harassment or discrimination, or where populist nationalist ideologies find fertile ground. These papers only begin to touch on the myriad of ways in which racial and ethnic inequality and political geography are intertwined, but we hope that they inspire continued future research in this direction. Second, though unintentionally, the papers in this Research Topic focus on European societies. To be sure, we would be the first to agree that the populist nationalist developments and racial and ethnic inequalities in European countries are important and interesting, but parallel developments in other parts of the world and especially in the Global South must also be the focus of future research, in this journal and elsewhere. Without explicit scholarly attention, it remains an open question how developments in different parts of the world are related to one another. We urge not only a wider range of individual empirical cases but also a more comparative and global lens on racial and ethnic inequality and populist nationalism. Finally, we would like to underscore the significance, in this current historical moment of upsurge in populist nationalist politics, of studying ethnic and racial inequalities. Across the articles in this collection, racial and ethnic inequalities are varyingly explanans and explanandum, and we encourage continued work to unpack the complicated ways in which ethnic and racial inequalities and populist nationalism are interrelated.

## Author contributions

CK developed and drafted the editorial. SC reviewed and edited the editorial. All authors approve of the final manuscript.

## Conflict of interest

The authors declare that the research was conducted in the absence of any commercial or financial relationships that could be construed as a potential conflict of interest.

## Publisher's note

All claims expressed in this article are solely those of the authors and do not necessarily represent those of their affiliated organizations, or those of the publisher, the editors and the reviewers. Any product that may be evaluated in this article, or claim that may be made by its manufacturer, is not guaranteed or endorsed by the publisher.

